# Complex Care: Schizophrenia Meets Rheumatoid Arthritis

**DOI:** 10.1192/j.eurpsy.2025.1207

**Published:** 2025-08-26

**Authors:** O. Laabidi, S. Laabidi, A. Touiti, N. Bram

**Affiliations:** 1 Forensic psychiatry department, razi hospital, la manouba, Tunisia

## Abstract

**Introduction:**

The management of coexisting schizophrenia and rheumatoid arthritis (RA) is complex due to the lack of clear guidelines. While immunosuppressants are the cornerstone of RA therapy and clozapine is the gold standard for treatment-resistant schizophrenia, their shared adverse effects make combined treatment challenging, requiring alternative therapeutic options.

**Objectives:**

present the therapeutic complexity of managing treatment-resistant schizophrenia alongside RA.

**Methods:**

We report a case of drug-induced toxidermia in a patient being treated for RA with some therapeutic particularities.

**Results:**

Mr. W, a 66-year-old man, has been under treatment since 2007 for schizophrenia. Somatically, he has rheumatoid arthritis (RA) that has been progressing since 2016, for which he is treated with Methotrexate.

The diagnosis of treatment-resistant schizophrenia was made. Given this resistance, the introduction of Clozapine was proposed. However, the issue of an interaction between Methotrexate and Clozapine, contraindicated according to pharmacologists’ report, required therapeutic reevaluation.

Clinical and biological evaluation showed remission of RA with an EVA score of 0, NAD of 0, NAG of 0, a DAS28 score of 1.13, and an VS of 5 mm. The choice then fell on Sulfasalazine to replace Methotrexate, with a progressive introduction of the drug, starting at 500 mg and increasing by 500 mg every 7 days up to a dose of 2000 mg/day, accompanied by weekly control tests.

Twelve days after the introduction of Sulfasalazine, at a dose of 1000 mg/day, the patient presented with a diffuse rash associated with erythroderma, facial and eyelid edema, an infiltrated, pruritic maculo-erythematous rash, confluent in places but sparing the palms and soles. Given the characteristics of the dermatological lesions and their timing, drug-induced toxidermia caused by Sulfasalazine was strongly suspected. Consequently, Sulfasalazine was discontinued, as well as the antipsychotic, and the patient was treated with topical skin treatments and two boluses of intravenous corticosteroids.

The initial biological workup was normal. However, seven days after stopping Salazopyrin, the patient became febrile with a temperature of 40°C, accompanied by erythematous pharyngitis, an elevated CRP of 45, monocytosis, and the presence of activated lymphocytes on the blood smear. Pharmacovigilance opinion: a DRESS syndrome was suspected, with a Regi score of 2. Two weeks later, the clinical course was favorable, with the complete disappearance of dermatological lesions and normalization of biological parameters.

**Conclusions:**

Managing treatment-resistant schizophrenia alongside rheumatoid arthritis poses a therapeutic challenge due to drug interactions. The case of Sulfasalazine-induced toxidermia highlights this complexity. Close monitoring and tailored treatment alternatives are crucial to minimize risks while ensuring efficacy.

**Disclosure of Interest:**

None Declared

